# The role of Mediterranean Diet in mental health in pandemic times

**DOI:** 10.1192/j.eurpsy.2021.1788

**Published:** 2021-08-13

**Authors:** A. Fraga, D. Esteves-Sousa, J. Facucho-Oliveira, M. Albuquerque, M. Costa, N. Moura, P. Espada-Santos, A. Moutinho

**Affiliations:** 1 Mental Health, Hospital de Cascais, Alcabideche, Portugal; 2 Psychiatry, Hospital de Cascais, Cascais, Portugal; 3 Psychiatry Department, Ocidental Lisbon Hospital Center, Lisboa, Portugal

**Keywords:** COVID-19, Depression, mental health, Mediaterranean Diet

## Abstract

**Introduction:**

In late 2019, an epidemic outbreak emerges in China caused by a new coronavirus with high transmission and human infection potential which in March 2020, was characterized by WHO as a pandemic. The lockdown has repercussions on the population’s well-being, reflected in their food choices. There is a tendency to increase the consumption of energy dense food, rich in fat and carbohydrates, which are related to an increased risk of depression.

**Objectives:**

The main goal of this non-systematic literature review was to understand the impact of the Mediterranean Diet on Mental Health promotion in SARSCoV-2 pandemic.

**Methods:**

Literature from Pubmed database were searched, with the following keywords: COVID-19, Depression, Anxiety, Mental Health and Mediterranean Diet.

**Results:**

Studies indicate that a diet based on the Mediterranean Diet is associated with a decreased risk of developing depressive symptoms, especially when there is moderate to high adherence to this dietary pattern. High consumption of plant and fish foods, reduced consumption of sugary products, processed and red meats and the use of olive oil as a fat source, are principles of the Mediterranean diet, associated with an improvement in endothelial function, increased levels of eicosanoids and serotonin synthesis and regulation of serotonin which seem to explain this protective effect.

**Conclusions:**

In addition to decreasing the risk of obesity, diabetes, and hypertension, comorbidities associated with the most serious disease of COVID-19, the Mediterranean Diet seems to play an important role in promoting mental health, with a decreased risk of developing depressive symptoms.

**Disclosure:**

No significant relationships.

